# A call to action for an equitable global genomic system

**DOI:** 10.1038/s41588-026-02667-y

**Published:** 2026-09-01

**Authors:** Deborah Mascalzoni, Sara Niedbalski, Tiffany Boughtwood, Kazuto Kato, Ciara Staunton, Michèle Ramsay, Anna Laura Ross, Ahmad Abou Tayoun

**Affiliations:** 1 Department of Public Health and Caring Science, Uppsala University, Uppsala, Sweden; 2 Institute for Biomedicine, Eurac Research, Bolzano, Italy; 3 Consultant to the World Health Organization, Geneva, Switzerland from November 2024 to July 2025; 4 Genomics Australia, Canberra, Australia; 5 Graduate School of Medicine and Premium Research Institute for Human Metaverse Medicine, The University of Osaka, Suita, Japan; 6 University of Modena and Reggio Emilia, Italy; 7 University of Kwazulu-Natal, South Africa; 8 Royal College of Science, Ireland; 9 Sydney Brenner Institute for Molecular Bioscience, Faculty of Health Sciences, University of the Witwatersrand, Johannesburg, South Africa; 10 World Health Organization (WHO), 20 Avenue Appia, 1211 Geneva, Switzerland; 11 Genomic Medicine Center, Dubai Health, Dubai, UAE; 12 Center for Genomic Discovery, Dubai Health, Dubai, UAE; 13 College of Medicine, Mohammed Bin Rashid University, Dubai Health, Dubai, UAE; 14 Member of the WHO Technical Advisory Group on Genomics (TAG-G)

## Abstract

As genomics infrastructure expands worldwide, the governance choices made now will determine whether genomics advance global health equity or entrench existing disparities, where today’s biased datasets will be locked into tomorrow’s clinical tools. This Comment advocates for inclusive, community-centered genomic systems, before biased data and extractive models becoming the default.

The rapid global expansion of genomic infrastructure presents a once-in-a-generation opportunity to embed equity, ethics, and public value into how human genomic data are collected, governed, and used. As genomics becomes increasingly central to health systems, biomedical research, and artificial intelligence–driven innovation, policy decisions made today on how genomics is funded and governed and how resulting tools are deployed will have downstream consequences and determine whose lives are improved, whose data and knowledge are recognized and represented, and whose needs remain invisible.

The World Health Organization’s (WHO’s) 2024 Guidance for human genome data collection, access, use and sharing governance (https://www.who.int/publications/i/item/9789240102149) offers a timely normative foundation, articulating principles of equity, sustainability, solidarity, and respect for rights (**Box 1**). These principles represent an essential first step. Yet, moving from principles to enforeceable practice requires intentional, stepwise implementation into governance frameworks institutionally, nationally and internationally–specifying responsible stakeholders, timelines, and accountability mechanisms. Otherwise, genomic systems risk reproducing historical inequities through exclusionary data practices, extractive research models, and the unchecked deployment of biased datasets in clinical and computational tools including research, diagnostic, and decision-support applications.

This Comment represents a call to action advocating for the implementation of a global agenda that encompasses different levels of governance and is directed towards national and international policy makers, funders, and ethics bodies, as well as the broader scientific community whose norms and practices shape actual governance. It highlights persistent current disparities in genomic data generation and access, the role of emerging national and regional initiatives, and governance models grounded in public trust and community engagement that could provide insights into potential directions. At a moment when public investment in science, inclusive research, and global collaboration is increasingly contested in parts of the world, reaffirming shared values and collective responsibility in genomics is not optional—it is essential. The recent endorsement of the WHA79.8 resolution on precision medicine by the 79^th^ World Health Assembly higlights the urgency of this call for action (https://apps.who.int/gb/ebwha/pdf_files/EB158/B158_R2-en.pdf).

## A narrowing window of opportunity

Genomics stands at a critical inflection point. Around the world, governments and research institutions are investing heavily in sequencing infrastructure, biobanks, and data platforms, while advances in artificial intelligence (AI) promise to amplify the reach, impact and clinical translation of genomic data. These developments create powerful incentives for rapid data accumulation and reuse—but also heighten the ethical stakes. Once genomic data pipelines, standards, and governance norms are entrenched, they become difficult to reverse.

At the same time, genomics is no longer confined to research settings. It is becoming embedded in routine health care and population-level infrastructures, particularly in oncology, rare disease diagnosis, pharmacogenomics, and genomic screening programs. As these applications expand, value-shaped policy becomes paramount to avoid discrimination, misrepresentation, and unequal access to emerging benefits.

Genomic governance norms are still being formed: data access and sharing policies, benefit-sharing arrangements, and consent frameworks remain contested and unsettled across jurisdictions (Table 1); this will directly influence who can access new diagnostics and therapies, and on what terms. If high-income, high-capacity actors continue to set these standards unilaterally, these will become entrenched through legal instruments, platform architectures, and institutional inertia—making subsequent reform progressively harder. This concern is not hypothetical; cross-border genomic governance already reveals significant disparities in negotiating power and capacity ^1^.

Acting now is not simply prudent—it is necessary to prevent existing inequities from being further entrenched and new ones from being built into the foundations of genomic medicine ^2^. This call is directed at policymakers setting national and international genomic strategies, funders designing grant requirements, researchers establishing data-sharing practices, international bodies developing technical standards and the wider researcher community that builds practices which translate into policies.

## A biased baseline

Despite decades of scientific progress, the genomic data landscape remains profoundly uneven. The majority of large-scale genomic datasets are still disproportionately derived from individuals of European ancestry, a failure of diversity, representation, and equity (**Box 2**), that limits discovery and undermines the equitable application of precision medicine.

Findings derived from currently biased genomic datasets often translate poorly across populations, leading to misclassification of variants, reduced risk assessment or diagnostic accuracy, biased therapeutic targets, and inequitable clinical outcomes. These gaps are especially visible in areas, such as rare disease and cancer genomics, where population-specific variants can significantly affect risk prediction and treatment response.

Without intentional correction, biased baselines risk becoming self-reinforcing. Data-driven tools trained on unrepresentative datasets can amplify disparities, particularly as genomics is integrated with AI systems used for diagnosis, prognosis, and clinical decision-making. Addressing representation is therefore foundational to genomic equity, scientific validity, and public trust.

## Global genomic initiatives and the pursuit of equity and public value

A new generation of national and regional genomic initiatives is rapidly reshaping the global landscape. Programs, such as Genome of Europe (https://genomeofeurope.eu/white-paper-published-by-genome-of-europe/), MyGenome Malaysia (https://mygenome.asia/), the Singapore Genome Project ^3^, as well as those in the Greater Middle East ^4^ and Africa ^5^ reflect growing recognition of the need for population diversity, local capacity building, and strategic investment in genomics on a global scale. These initiatives offer genuine opportunities to broaden representation, strengthen scientific ecosystems, and support regionally relevant research and health applications.

At the same time, they raise complex questions about data governance, identity, access, and public value. In the absence of shared ethical standards and interoperable governance frameworks (Table 1), fragmented or competitive approaches may deepen global disparities rather than reduce them, and will continue to risk inequitable access to genomic-based innovations.

A crucial conceptual distinction must be made here between normative consensus and implementation pluralism. Normative consensus refers to shared acceptance of foundational global principles—such as those in the WHO 2024 guidance—that establish non-negotiable ethical baseline. Implementation pluralism refers to the legitimate variation in how those principles are operationalized across diverse political, economic, and cultural contexts. The goal is not to impose a single governance model, but to ensure that diversity in implementation does not become a vehicle for circumventing shared ethical commitments, ensuring these initiatives advance not only local interests, but also public value, equity, and global health solidarity. Coordination, transparency, and mutual accountability will be essential if genomic infrastructure is to serve collective rather than narrowly defined goals. This requires translating values into workable approaches that embed shared values by design without imposing a single ethical model. Federated data access and novel sharing approaches (**Box 3**) accommodate diverse understandings of data governance allowing different actors to enable local policies and specific social norms, required by different communities, while maximizing the availability of data on a global scale.

## Lessons in sovereignty and solidarity from Indigenous genomics

Some of the most instructive lessons in genomic governance come from Indigenous and historically marginalized communities. Experiences from Aboriginal, Native, and First Nations groups have exposed the limitations of conventional research models that prioritize extraction over partnership and individual consent over community control. In response, many communities have articulated alternative approaches centered on data-sovereignty, stewardship, and cultural context demanding Indigenous-developed and led research to support community’s self-determination and reduce harm from research findings^12^.

Frameworks, such as the CARE (Collective benefit, Authority to control, Responsibility, and Ethics (https://www.gida-global.org/care) or the OCAP Principles ^6^ and UN declaration on the rights of indigenous people, emphasize that data governance must reflect community values and lived realities. These approaches challenge dominant paradigms by insisting that participation in genomic research does not require relinquishing control or identity. Respecting data-sovereignty can coexist with global collaboration, provided governance systems are designed to support pluralism, reciprocity, and trust (see **Box 3**). These lessons are broadly applicable as genomics expands into diverse populations worldwide.

## Building equitable systems from principles to practice

Translating normative principles into global practice and tools requires deliberate institutional design that extends beyond national borders. Using the WHO guidance as a general framework, equity, solidarity, sustainability, and rights can only be operationalized through policies that enforce inclusive data collection, transparent governance, and meaningful public engagement. Governance models (see **Box 3**) must play a central role in rebalancing power toward participants, communities, and public institutions—for example through collective decision-making structures, differentiated access, and conditions for data access and use that reflect public priorities.

As genomics expands, there is a growing risk that “engagement” becomes performative unless communities have genuine influence over decision-making, including priority setting, governance design, and, where appropriate, the ability to withhold consent or exercise veto rights. Emerging models of governance should be built in partnership with communities, and should entail adaptive consent mechanisms, as well as dynamic oversight that evolves alongside technology. Sustainability must also be understood as a governance challenge, not just a financial or technical one. Investments in sequencing capacity, data platforms, and analytics will not deliver equitable outcomes in the absence of durable governance frameworks that can evolve over time, manage competing interests, and ensure accountability.

International bodies can provide a general framework, and a push to develop local guidance that embraces shared basic values (**Box 4**). National governments play a critical role but they are not the only actors. Research funders, ethics bodies, professional scientific societies, journal editors, and international bodies each carry specific responsibilities in setting regulatory expectations, funding inclusive infrastructure, supporting capacity building, particularly in low- and middle-income countries, aligning genomics with public health priorities and addressing equity and benefit-sharing nationally and across borders. At the practical level, regional collaborations could serve as a forum as well as guidance for translating principles into practical changes. For example, stakeholders of genomics within each of the six regions of the WHO, have been discussing how to promote equitable access to genomic technology for public health in each region (**Box 4**).

Equitable systems do not emerge by default. They require sustained investment, interdisciplinary expertise, and a willingness to redistribute power within research ecosystems. Equity should be treated not only as an ethical aspiration but also as a measurable precondition for legitimacy and uptake ^7^. The development and use of equity and public value indicators, such as representation across populations, access to downstream benefits, and distribution of risks, can support accountability and guide adaptive governance as genomic applications scale (**Box 5**).

## The risks of delay

Failing to act at this juncture now carries significant risks. Bias embedded in genomic datasets today will shape AI-driven tools tomorrow, potentially entrenching disparities in diagnosis, treatment, and resource allocation ^8^. AI systems trained on non-representative genomic data are already producing differential clinical recommendations across population groups, and the pace of AI deployment in clinical genomics means that governance gaps compound rapidly. Weak governance can erode public trust, undermining participation and long-term sustainability.

These risks are amplified by a broader political context, in which public investment in science, global cooperation, and inclusive research is increasingly under strain. Policy shifts that deprioritize equity, restrict inclusive language, or defund global health initiatives threaten to normalize exclusion. Without sustained political and institutional commitment, genomic infrastructure may reproduce the very inequalities it promises to address.

## Conclusions

The question facing the global genomics community is not whether genomic data will shape the future of health, but which values will be encoded in that future. Governance choices made now will thus determine whether genomics becomes a force for shared benefit or a driver of deepening inequality.

The research community has a specific role to play: adopting equity-reporting standards, including measurement by design, designing benefit-sharing from the outset, building community co-production into research practice, investing in under-resourced capacity, and engaging actively with governance processes rather than treating them as constraints on scientific progress.

A future worth building requires renewed global commitment to solidarity, accountability, and public value. Shared frameworks, inclusive governance, and respect for diversity and human rights are not obstacles to innovation—they are its very foundation. By acting decisively and collaboratively, the global community can ensure that the benefits of genomics are equitably governed in the service of global health.

## Figures and Tables

**Table 1:** Governance and operational differences among genomic initiatives worldwide

Initiative	Community consent	Benefit-sharing	Data access tiering	Interoperability	Enforcement	Equity ii leaders)
H3Africa https://h3africa.org/	Strong community engagement protocols	Partial; benefit-sharing principles stated	Tiered access implemented	GA4GH-aligned	Limited; no binding enforcement	African principal investigator leadership required
Genome of Europe https://genomeofeurope.eu/white-paper-published-by-genome-of-europe/	National consent models; heterogeneous	Not explicitly addressed	Federated access	GA4GH/1+MG aligned	EU regulatory framework	European-led; governance under construction
1+MG https://digital-strategy.ec.europa.eu/en/policies/1-million-genomes	Consent heterogeneous across member states	Not addressed	Federated/ processing environments	GA4GH aligned	EHDS regulation (in development)	EU member states; partial
MyGenome Malaysia https://mygenome.asia	National consent framework	Under development	Controlled access	Partial	National oversight	Malaysian-led; partial equity provisions
Canadian Precision Health Initiative https://genomecanada.ca/challenge-areas/canadian-precision-health-initiative/	Standard consent	Contested re: Indigenous communities	Controlled access	Partial	National	Indigenous leadership contested
All of Us (US) https://allofus.nih.gov/	Tiered consent with community advisory boards	Community return of results; limited	Controlled access tiers	Partial GA4GH	NIH oversight	Diverse recruitment goals; partial equity
gnomAD https://gnomad.broadinstitute.org/	Aggregated/summary; original consent via contributing studies	Open access; no individual benefit-sharing	No individual-level access	High interoperability	No formal enforcement mechanism	Global contributors; limited governance structure

## Box 1 Core principles of the WHO Guidance for Human Genome Data Collection, Access, Use and Sharing (2024)

The WHO 2024 guidance (https://www.who.int/publications/i/item/9789240102149) is chosen here over alternative frameworks for specific reasons. Built through a co-creative process that involved all 6 WHO regions with representation from 194 member states, it is meant to complement existing guidelines and provide a direction for new legal, ethical and policy architectures, grounding them through an ethical lens across geographies. The impact will depend on how it is operationalized across diverse political, economic, and cultural contexts. The Guidance articulates eight interconnected principles that together address the a high level spectrum of genomic governance challenges

1. **Affirming the rights of individuals and communities to make decisions**. A commitment to affirm and value the rights and interests of individuals with capacity to make informed decisions about their human genome data throughout the data life cycle, and to support those without such capacity. Because genomic data carry implications for families and communities, the views of family members and communities must also be taken into account. This principle also tackles the power asymmetries that arise when individual consent models are used as a substitute for genuine community engagement.
2. **Social justice**. A commitment to uphold individual and collective values and to enable the collection, access, use and sharing of human genome data in ways that promote the highest attainable standard of health and well-being, and that address the needs of underserved and marginalized individuals, families and communities. Fulfilling this commitment requires greater effort by promoting policies leading to equitable access, and others preventing discrimination and bias.
3. **Solidarity**. A commitment to stand in solidarity with others by ensuring equitable access to human genome data and fair distribution of its benefits and burdens, within and across communities, countries and regions. This principle acknowledges differences in capacity and existing inequities, and underpins the duty to share both the gains and the responsibilities of genomic science.
4. **Equitable access to and benefit from human genome data**. A commitment to achieve equitable collection, access, use and sharing of human genome data and its resulting benefits, by actively addressing power imbalances among stakeholders, increasing diversity and representation in datasets and among decision-makers, and ensuring that contributing individuals, families and communities fairly benefit. This principle directly targets disparities and inequities at the individual, organisational and international levels.
5. **Collaboration, cooperation and partnership**. A commitment to foster cross-border, cross-sector and cross-disciplinary collaboration in human genome data activities, including capacity building, knowledge transfer and joint governance arrangements. This principle directly addresses the structural asymmetries between high-capacity and low-capacity actors that this Comment identifies as the deepest challenge in global genomics.
6. **Stewardship of human genome data**. A commitment to responsible custodianship throughout the data life cycle: ensuring scientific quality, integrity and reproducibility; protecting privacy and security; preventing re-identification, surveillance and misuse; and maintaining the long-term value of the data for individuals, communities and science. This principle is foundational to building the public trust necessary for equitable participation.
7. **Transparency**. A commitment to ensure that processes for the collection, access, use and sharing of human genome data are open, accessible, understandable and ongoing — including how decisions are made, who benefits, what risks arise, and how data are stored, transferred and reused (including for AI training). Transparency is a precondition for meaningful consent, informed community engagement and credible governance.
4. **Accountability**. A commitment to clearly identify responsibilities and duties for all those involved in the human genome data life cycle, with effective oversight, redress mechanisms and proportionate sanctions for non-compliance. Accountability operationalises every other principle and is what distinguishes aspirational governance from enforceable governance.

## Box 2

Addressing consistently lack of diversity and representativeness ^9^, across governance levels, has a direct link to equitable approaches in genomics, given the fact that systematic exclusion of certain groups lead to structural inequity to access, and even to develop knowledge and infrastructures for genomics in LIMC; systematic underrepresentation is part of a set of power asymmetries, that if not seriously confronted will produces systems that embed them as part of an accepted inequal system. We provide distint definitions for the “representativeness”, “diversity”, and “equity” terminologies:

*Representativeness:*

- refers to statistical sampling adequacy including sex/gender/ age
- Reflects whether a dataset represents the demographic composition of the population it describes

*Increase Diversity:*

- meaningful inclusion of historically excluded groups or ancestries
- lack of diversity leads to disparities (empirical inequalities in data diversity) that in turn lead to inequities in access to research results and ultimately to health results; this underrepresentation reflects structural, not merely technical, failures

*Increase Equity:*

- refers to structural fairness in access and benefit — the distribution of opportunities, resources, and outcomes
- Power asymmetries challenge equity, and this aspect should be addressed at different governance levels

## Box 3 A pluralistic global genomics framework

We highlight three governance levels: (1) Institutional-level governance—consent and benefit-sharing arrangements within individual Institutions or biobanks; (2) national-level governance—legal frameworks regulating data collection, access, and sharing within jurisdictions; and (3) global-level governance—international standards, multilateral agreements, and interoperability norms that coordinate across jurisdictions. We argue that all three levels are necessary and that they interact: local projects are more likely to adopt equitable practices when national frameworks require it, and national frameworks are more likely to converge when anchored in a credible international standard—the role we ascribe to the WHO 2024 guidance.

Genomics frameworks must accommodate diverse cultural, ethical and governance perspectives rarther than impose a uniform “one size fits all approach”. Embracing a pluralistic approach means to recognize that different communities may hold legitimately different values regarding data ownership, sharing, and use, and that governance systems must be designed to accommodate this variation rather than override it. Reciprocity refers to the obligation of research systems to return tangible value to communities whose data and participation make research possible. Absence of “pluralism governance structures” risks perpetuating existing power imbalances where the views of the most powerful stakeholders dominate decision-making processes. So far, the narrative of the need to share has led to very specific approaches to push open science in a way that mostly benefited the Global North where extractive models excluded communities from the decision table.

There is convincing advocacy ^10^ for establishing a new “social contract” for genomic data sharing grounded in participatory governance models that incorporate equitable benefit distribution. Indigenous governance frameworks articulate how to achieve these goals by centering community values ^6^. These frameworks ensure that while data access continues, it operates through mechanisms that prioritize community authority, enable meaningful participation in decision-making processes, and align data use with community interests and priorities. The Silent Genomes project ^11^ (https://www.bcchr.ca/silent-genomes-project) exemplifies this approach by addressing the genomic divide affecting Indigenous populations while simultaneously developing community-based governance structures. Solidarity-based governance reflects core principles found in Indigenous models by establishing clear parameters that define public value boundaries, evaluating the distribution of burdens, benefits, and responsibilities throughout the data lifecycle, and providing practical frameworks for equitable sharing of both harms and benefits.

Current governance models take several distinct forms: centralized national repositories (where a single national institution holds and controls access to data); federated data-access systems (where data remain distributed but are made queryable under harmonized protocols; community-governed biobanks (where governance authority rests primarily with contributing communities); and hybrid public-private structures (where commercial entities operate within regulatory frameworks set by public bodies). Each model carries distinct implications for power distribution, accountability, and equity in access to benefits.Governance models for accessing data could potentially be a federated resource with a unique entry point to maximize access to datasets with diversified governance models within, as in some existing examples: the Genome Aggregation Database, gnomAD) initiative, (https://gnomad.broadinstitute.org/) the Pan Canadian Genome library(https://genomelibrary.ca/) ^12^ and EGA (https://ega-archive.org/about/projects-and-funders/federated-ega/).

The Federated European Genome-phenome Archive (FEGA) is a distributed infrastructure for securely sharing sensitive human omics data across national borders while keeping datasets stored locally within their country of origin. The architecture consists of a Central EGA that manages master infrastructure and standards, nationally funded FEGA Nodes that physically store datasets and manage local access requests, and FEGA Affiliates that independently archive and control their own data while contributing to the global discovery catalog. Data remains under Controlled Access, with data providers retaining authority to approve requests, and approved researchers can securely analyze data in Trusted Research Environments without transferring raw data across borders. This model respects strict national data protection laws while enabling global scientific collaboration and discovery (https://ega-archive.org/about/projects-and-funders/federated-ega/).

## Box 4 First steps

At the regional level, stakeholders within each of the six WHO regions are developing context-specific approaches to equitable genomic governance, and their experiences offer models for translating principles into practice (see also Table 1). In the WHO African Region (AFRO), initiatives, such as H3Africa and the African Genomics Initiative, have established important foundations for community-engaged, African-led genomic research, though governance gaps in enforcement and benefit-sharing persist. In the Region of the Americas (AMRO/PAHO), the PAHO genomics portal provides regional coordination for population genomics and health equity objectives. In the South-East Asia Region (SEARO) and Eastern Mediterranean Region (EMRO), genomic governance frameworks remain at earlier stages of development, but national programs are emerging with increasing momentum. In the Western Pacific Region (WPRO), an expert meeting convened in November 2024 identified regional priorities for equitable genomic data governance. In the European Region (EURO), the 1+MG initiative and the European Health Data Space are pioneering federated governance models that balance data availability with privacy and equity, though challenges related to benefit-sharing and non-EU participation remain unresolved.
